# Analysis of Dental Malocclusion and Neuromotor Control in Young Healthy Subjects through New Evaluation Tools

**DOI:** 10.3390/jfmk4010005

**Published:** 2019-01-14

**Authors:** Barbara Isaia, Martina Ravarotto, Paolo Finotti, Matteo Nogara, Giovanni Piran, Jacopo Gamberini, Carlo Biz, Stefano Masiero, Antonio Frizziero

**Affiliations:** 1 Studio Dentistico Isaia, Vigodarzere, 35010 Padua, Italy; 2 Department of Physical and Rehabilitation Medicine, University of Padua, 35128 Padua, Italy; 3 Orthopaedic and Traumatology Clinic, Department of Surgery, Oncology and Gastroenterology DiSCOG, University of Padua, 35128 Padua, Italy

**Keywords:** dental occlusion, malocclusion, stomatognathic system, posture, body balance, neuromotor control

## Abstract

The presence of a correlation between stomatognathic and postural systems has been investigated by different authors trying to identify a possible influence of dental occlusion on body posture and balance. The aim of this study was to evaluate the relationship between dental occlusion and neuromuscular control in a healthy young population using modern evaluation tools. 25 subjects (9 males and 16 females, aged 23 to 44) were evaluated for dental occlusion, particularly in relation to overjet and overbite parameters, anterior and posterior crossbite, scissor bite, mandibular crowding, molar and canine class, and deviation of the median dental line. Neuromotor control was assessed using two different types of stabilometric platforms in both monopodalic and bipodalic equilibriums (Prokin-B and MF-Stability, Tecnobody, Italy). All subjects were evaluated with and without cotton rolls positioned between the upper and lower arches at the premolar level in order to temporarily eliminate any pathological contact. In all 25 subjects, no statistically significant differences were revealed between the evaluations performed with and without cotton rolls in all the analyzed conditions (in static, in dynamics, with open and closed eyes). This study did not find a significant correlation between dental occlusion and neuromuscular control in a young and healthy population.

## 1. Introduction

Posture and its control has been frequently investigated by many specialists who have tried to give a precise definition of correct posture both anatomically and functionally, leading to the development of a specific branch of study called posturology. The term “posture” defines the position of the human body in space and the subsequent relationship between its segments. Posture can be defined as correct when it allows for implementing any movement with the least energy expenditure, being characterized by the absence of asymmetric or abnormal muscular tensions and by correct relationships between the various body segments [1,2].

Posture is not a single entity for each subject, but it refers to any “balanced position”, defined with maximum equilibrium (stability), economy (minimum energy consumption), and comfort (minimum stress on anatomical structures). Postural regulation requires the control of the so-called “Tonic Postural System”, which is a cybernetic system characterized by a constant transmission of information between the locomotor apparatus and central nervous system (CNS). The latter receives all information from exteroceptive (tactile, visual, and auditory) and proprioceptive receptors, processes it, and sends response outputs to the end effectors. Each component of this system can sometimes be altered, thus modifying body balance control [3,4]. In the last few years, thanks to technological innovations, knowledge on this topic has made big steps forward, leading to the awareness that posture is involved in many musculoskeletal problems [5,6,7,8,9].

Particular attention has been placed on occlusal relationships, trying to understand whether an altered dental occlusion can lead to changes in muscular patterns. Various studies have shown that changes in mandibular position can induce variations in postural settings, showing the existence of a biomechanical and neurological connection between stomatognathic and postural systems [10,11]. The stomatognathic system is an anatomic-functional complex that performs digestive, respiratory, relational, and postural functions. Indeed, the skull, jaw, and cervical spine constitute an inseparable functional unit, so that everything that happens in the mouth has an impact, through temporal-mandibular joints, on the cervical tract, thus affecting the scapular girdle, the vertebral column up to the feet, and vice versa. For this reason, stomatognathic dysfunctions can cause cranium-cervical-mandibular alterations that in turn can generate postural imbalances [12].

**Figure 1:**
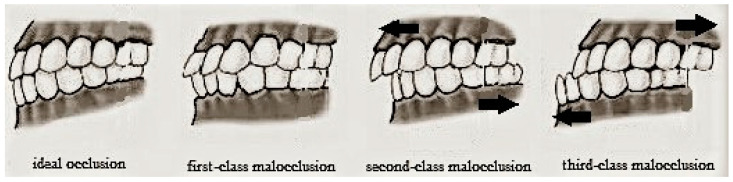
Angle classification of dental occlusions. Normal occlusion: The first upper molar is displaced distally from the first lower molar, for no less than half a cusp. First-class malocclusion: The relationship between molars is normal but the alignment of other teeth is not. Second-class malocclusion: The jaw is positioned farther back than the maxilla, so that upper incisors show accentuated protrusion and overjet. Third-class malocclusion: The jaw is too advanced in relation to the maxilla. (Figure 1 is inspired by the image in the following link: https://img.tfd.com/dorland/malocclusion.jpg).

**Figure 2:**
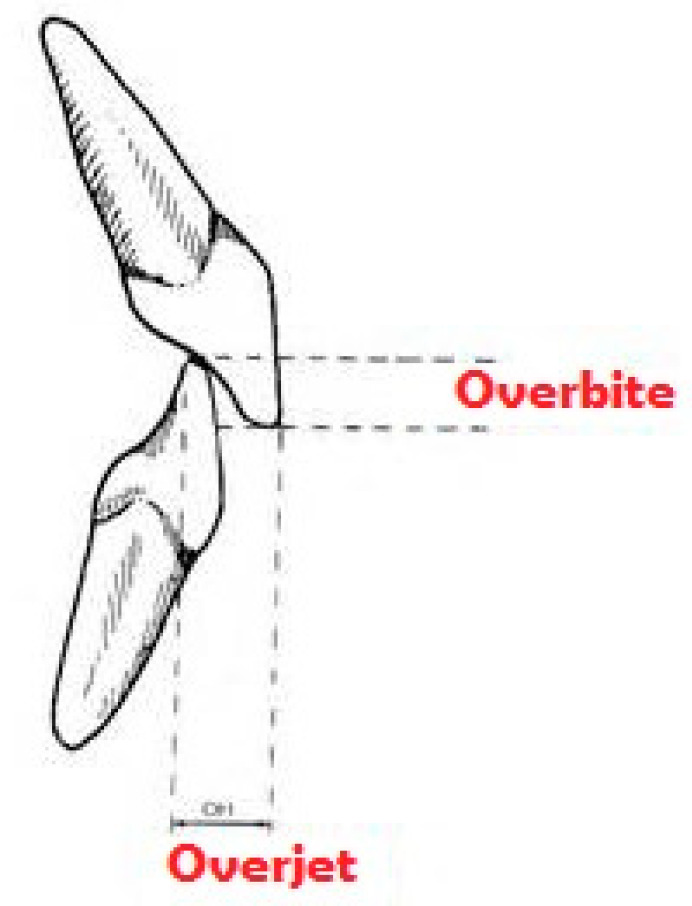
Representation of overjet and overbite parameters. Overjet refers to the horizontal distance between the edge of upper and lower incisors, while overbite represents the vertical distance between the free margins of the same elements.

The term “occlusion” defines the relationship between maxillary and mandibular arches, when teeth come into contact with each other both in static conditions and during functional movements of the temporomandibular joint. This contact should always be uniform and simultaneous on both sides, in order to give the jaw maximum stability using as many contacts as possible. Changes in normal occlusal morphology may induce the jaw to look for a stable occlusion position, which may not correspond to that in which the chewing muscles and internal structures of the temporomandibular joint are used to operating in normal conditions [13]. In orthodontics, all non-ideal occlusal situations are identified as “malocclusions”, and they are usually classified into three classes, according to Angle’s classification (Figure 1) while the occlusal relationship between anterior dental elements is defined by overjet and overbite parameters (Figure 2). Each dental malocclusion can cause repercussions on the whole postural system [14].

In literature, several studies have identified a correlation between stomatognathic and postural systems, demonstrating that different mandibular positions can induce variations in body posture [15,16,17]. In 1976, Funakoshi et al. analyzed the relationships between changes in head posture and the functioning of chewing muscles through electromyography investigation, showing that an occlusal interference could result in an incorrect reaction of masseter and temporal muscles, depending on the head position [18]. In 1992, Martensmeier et al. showed the radiographic modifications of cervical spine curvature in the sagittal plane following orthodontic treatment for malocclusion [19].

In their work, Tardieu et al. studied the influence of a dental occlusion perturbation on postural control. The tests were performed in three dental occlusion conditions (rest position, maximal intercuspal occlusion, and thwarted laterality occlusion, which represented a simulation of a dental malocclusion) and four postural conditions (static and dynamic, with eyes open and eyes closed). Their results have proven that dental occlusion compromises postural control only in dynamic conditions and in the absence of visual signals; in fact, sensory information related to dental occlusion appeared to become effective only during difficult postural tasks, with a growing importance when other sensory signals were poor [20].

In contrast to the aforementioned studies, other works in literature documented the absence of a correlation between stomatognathic and postural systems, evaluating different parameters (equilibrium with closed and open eyes, loading distribution on feet, body oscillations) and showing no significant influence of dental occlusion on body posture [21,22].

The purpose of this study was to evaluate the presence of a possible association between dental malocclusions and body posture and balance in a healthy young population using modern evaluation tools.

## 2. Materials and Methods

### 2.1. Study Sample

The study sample was obtained from doctors who were specializing in Physical and Rehabilitation Medicine and Orthopedics at the University of Padua. The study group was composed of 25 subjects, aged 23 to 44 years, who voluntarily accepted to participate in the study. All subjects were asked if they had sustained dental or face injuries and if they had received orthodontic treatment with braces.

### 2.2. Subject Examination

For each subject, the evaluation consisted of an earlier clinical examination of the facial district, followed by an instrumental stability examination.

The facial examination was performed in frontal and profile views with the subject both at rest and while smiling, evaluating different parameters.

**Figure 3:**
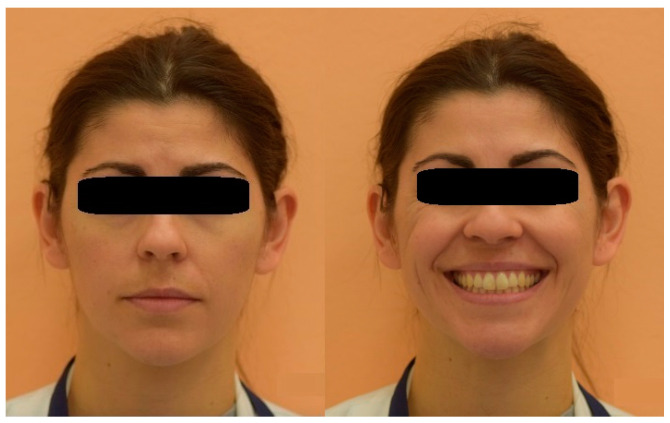
Facial examination in frontal view.

In the frontal view (Figure 3), the relationship between the eyes–nose–mouth and the facial symmetry were evaluated. In particular, the relationship between the midlines of dental arches and those of skeletal and soft tissue were considered. The vertical reference axis of the face was considered to be the one passing through the tip of the nose, the upper and lower midline of incisors, and the chin midpoint, while the bipupillar axis represented the horizontal reference axis. Any deviation from the midline of such parameters was considered as an asymmetry.

The height/width ratio of the face was also measured, with the height being represented by the distance between the hairline and the lowest point of the jawline, while the width was represented by the distance between the two most lateral points of the zygomatic arches. In a normal population, this ratio has a value around 1.3 for females and 1.35 for males.

**Figure 4:**
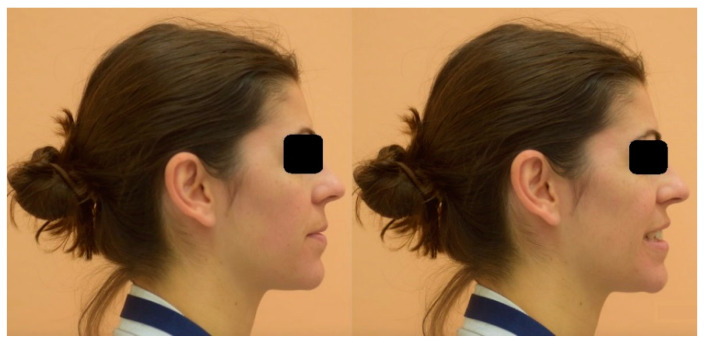
Facial examination in sagittal view.

In the profile view (Figure 4), two lines were used to identify the face profile—the first from the glabella to the upper lip margin, and the second from the upper lip margin to the chin. The angle between these two lines indicates a class II skeletal relationship if the profile is convex, or a class III relationship if the profile is concave.

Regarding dental occlusion, each subject was asked about previous orthodontic treatment and previous teeth or face trauma. Clinical examination was focused on different parameters: molar and canine classes, overjet, overbite, anterior and posterior crossbites, dental crowding, and deviation of the upper and lower dental midline. Molar and canine classes were described using Angle’s classification of malocclusions (Figure 1). Overjet is the horizontal (anterior–posterior) gap between the labial surface of the upper central incisors and the vestibular surface of the lower central incisors. Overbite is the vertical distance between the incisal edges of the upper and lower incisors (Figure 2).

### 2.3. Instrumental Assessment of Neuromotor Control

**Figure 5:**
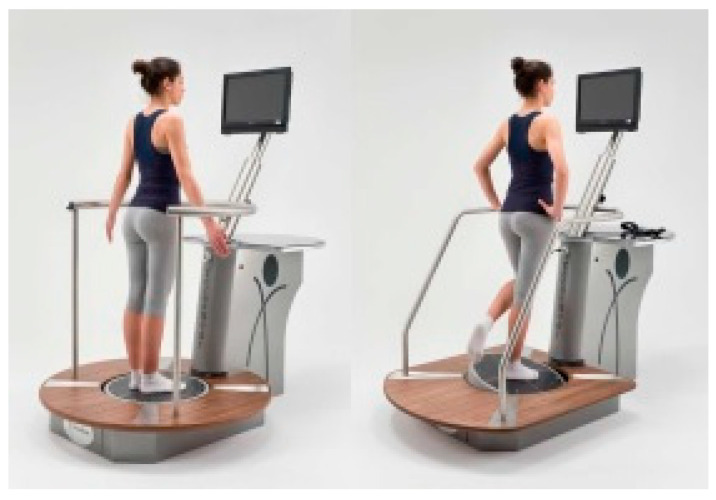
MF-Stability (**left image**) and Prokin (**right image**) devices, Tecnobody SRL, Italy.

Stability evaluations were performed at the Department of Orthopedic Rehabilitation of the University Hospital of Padua, using modern evaluation tools (Prokin-B and MF-Stability, Tecnobody SRL, Dalmine, Bergamo, Italy, Figure 5). Both Prokin-B and MF-Stability have two different difficulty levels (“standard” and “advanced”); for our sample, the “advanced” mode was chosen. Each evaluation was completed on the same day.

The subjects were asked to perform different tests in various conditions; in particular, the same test had to be performed with and without cotton rolls, which were placed between antagonist teeth because they were expected to remove occlusal interferences and mandibular slides along the physiological pattern of the jaw-closing movement [7].

The assessments were randomized so that some subjects performed each test before without cotton rolls and then with them, while other subjects did the contrary, in order to avoid potential bias due to a learning effect.

The MF-Stability device is equipped with a stabilometric platform and evaluates body posture in static conditions, involving the execution of different tests:

- A stabilometric test with open eyes (Romberg’s test) that consists of keeping on the standing station for 30 s while staring at a red spot on the monitor, during which it assesses the position of the body in space. The numerical value obtained is represented by the area described by the center of mass displacements (in mm^2^); the higher the value, the worse the test result.
- A test for the evaluation of the stability limit, in which the task is to move the center of gravity towards a point indicated in the monitor; this test is useful for evaluating spatial exploration capacity. The value obtained represents the displacement of the center of mass, expressed as a percentage of the total displacement provided by the software according to the height of the subject (more than 75% is considered normal).
- Two comparative stability tests, including a bipodalic test with open and closed eyes, and a monopodalic test comparing the balance between the right and left foot. The scores are expressed as the area described by the center of mass displacements (in mm^2^); the higher the value, the worse the test result.

Prokin-B is a device that evaluates the dynamic balance, as the subject has to perform three tests on an unstable platform:

- The first test consists of maintaining the bipodalic equilibrium on the unstable platform with visual feedback, which is useful for identifying the directions of the subject’s imbalance. The value provided by the device is the stability index, that is the distance (expressed in cm) between the center of the platform and the average coordinates of the positions assumed by the center of mass; lower scores correspond to greater stability.
- A second comparative proprioceptive test gives information about body control whilst performing a fine motor skill, in this case represented by tracking a circular trace on the monitor, either with the right foot (clockwise) or with the left foot (anti-clockwise). The result of this test is expressed as an “average trace error”, which indicates the deviation of the subject from the optimal trace, expressed as a percentage; a low value corresponds to a trace close to the ideal line, thus meaning good motor and perceptive capacity.
- A third comparative monopodalic equilibrium test consists in maintaining the dynamic equilibrium in a monopodalic position, first with the right foot and then with the left. The result is expressed as a stability index—that is, the distance (in cm) between the center of the platform and the average coordinates of the positions assumed by the center of mass; the lower the score, the greater the stability.

### 2.4. Statistical Analysis

For the statistical analysis procedure, since no evaluated subjects reported significant malocclusion, a comparison of data was performed between the normal condition and the state with cotton rolls placed between the dental arches. The results obtained with and without rolls were compared through the Wilcoxon signed-rank.

## 3. Results

### 3.1. MF-Stability

• Open-eyes stability test with and without cotton rolls

**Figure 6:**
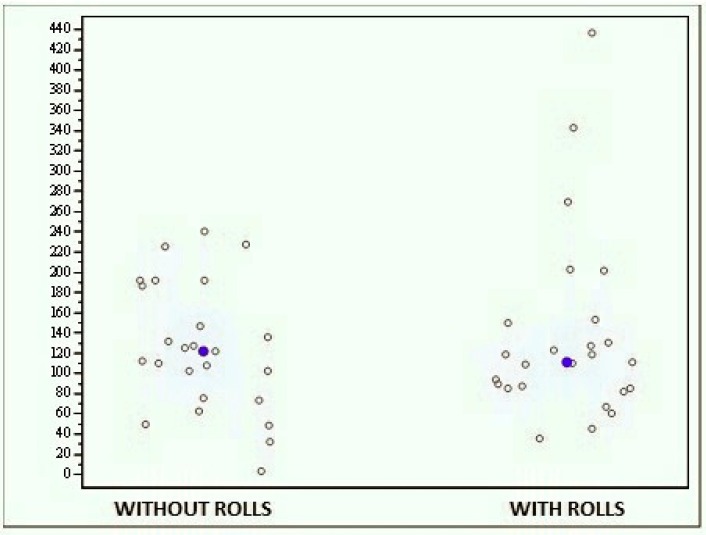
Graph showing the scores obtained with the MF-Stability device at the open-eyes stability test with and without cotton rolls.

The whole sample of subjects did not exhibit statistically significant differences between the results obtained with and without rolls, since the average score obtained without rolls was 125.088 (±63.737) while that obtained with rolls was 137.495 (±92.666) (Figure 6). Therefore, there were no statistically significant differences between the two tests (*p* = 0.7048).

• Open-eyes stability limit test with and without cotton rolls

**Figure 7:**
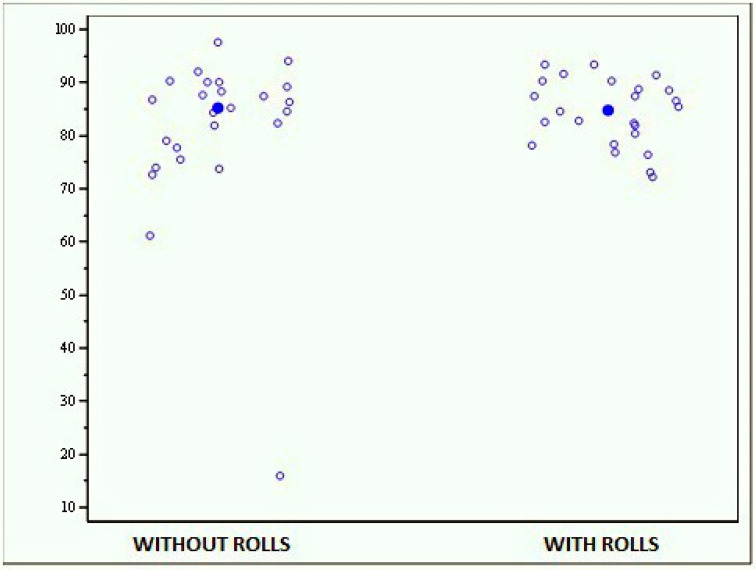
Graph showing the scores obtained with the MF-Stability device at the open-eyes stability limit test with and without cotton rolls.

The average scores reported by the whole sample were 81.124 (±15.787) for the test without rolls and 84.364 (±6.094) for that with rolls, with a test median, respectively, of 85.170 and 84.770 (Figure 7). These results show no statistically significant differences between the two evaluations (*p* = 0.4386).

• Monopodalic evaluation with and without rolls

**Figure 8:**
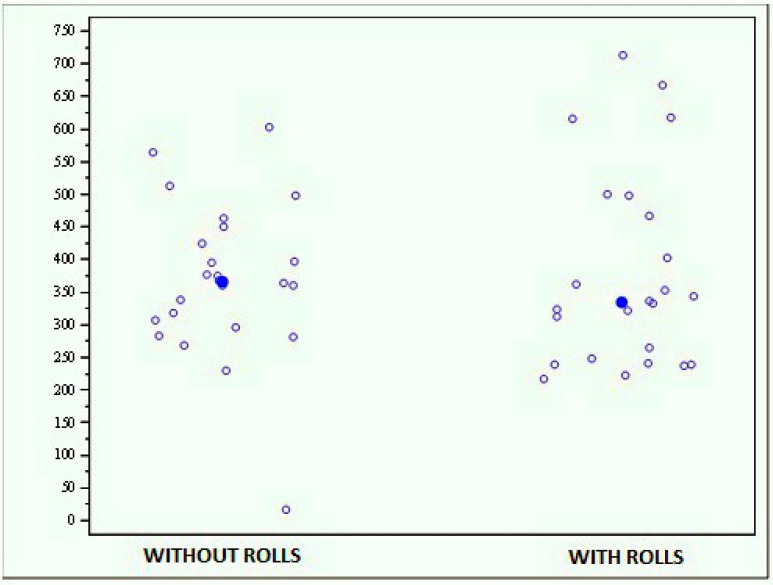
Graph showing the scores obtained with the MF-Stability device at the monopodalic evaluation with and without rolls.

The whole sample exhibited no significant differences between the results obtained with and without rolls (*p* = 0.7187); the average score of the evaluation performed without rolls was 368.729 (±119.993), while that of the evaluation performed with rolls was 378.385 (±150.530) (Figure 8).

Moreover, no significant differences have been found between the tests performed with the left or right foot.

• Bipodalic evaluation with open and closed eyes, with and without rolls

**Figure 9:**
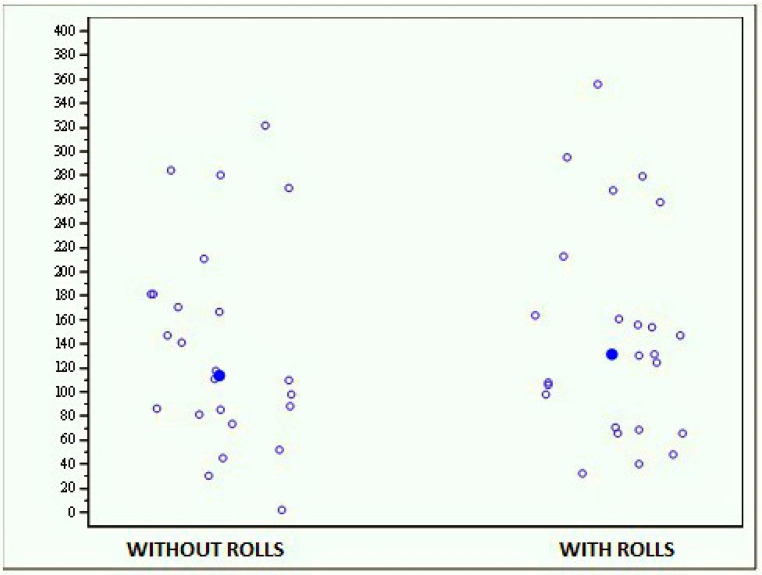
Graph showing the scores obtained with the MF-Stability device at the bipodalic evaluation with open eyes without and with rolls.

The results show that the subjects had better balance when they performed the test without rolls, even if this difference is not statistically significant (*p* = 0.5980). Indeed, the open-eyes test performed without rolls reported scores ranging from 2.250 to 321.410, while the same test performed with rolls reported scores ranging from 32,710 to 356,010 (Figure 9).

**Figure 10:**
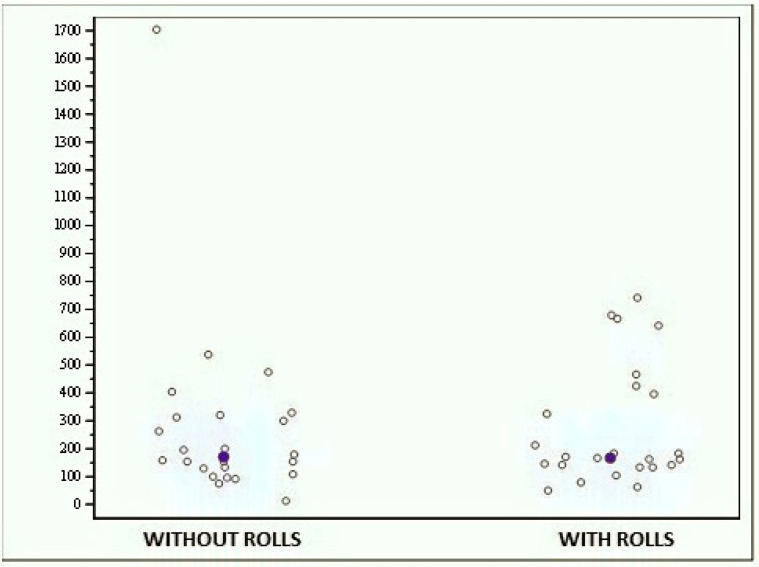
Graph showing the scores obtained with the MF-Stability device at the bipodalic evaluation with closed eyes without and with rolls.

At the bipodalic evaluation with closed eyes, the scores ranged from 11.890 to 1703.030 when the subjects performed the test without rolls and from 48,840 to 740,290 when the test was performed with rolls (Figure 10); even in this case, no significant difference was found (*p* = 0.287).

### 3.2. Prokin-B

• Bipodalic stability test with and without rolls

**Figure 11:**
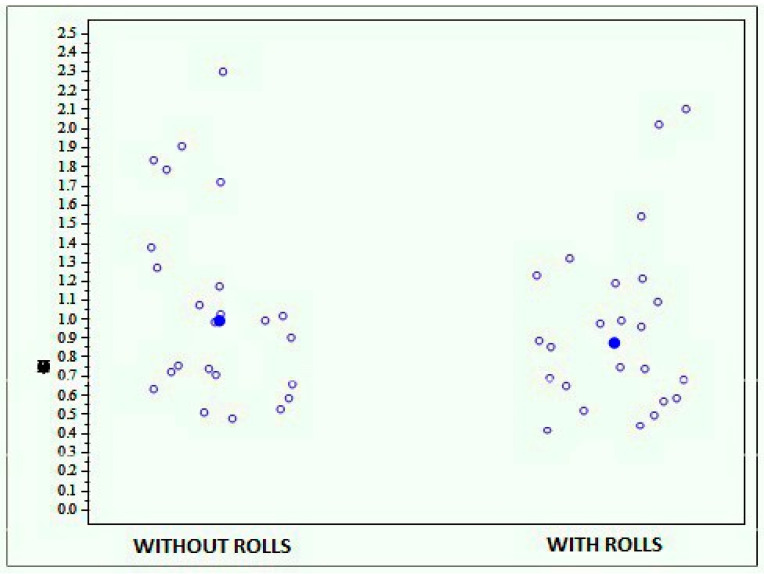
Graph showing the scores obtained with the Prokin-B device at the bipodalic stability test with and without rolls.

The results obtained by the whole sample show more stability in the test performed with rolls than in the one performed without rolls, reporting an average score of 1.072 (±0.508) with rolls and of 0.955 (±0.452) without rolls (Figure 11). However, this difference is not statistically significant (*p* = 0.1179).

• Proprioceptive evaluation through foot-ankle circling with and without rolls

**Figure 12:**
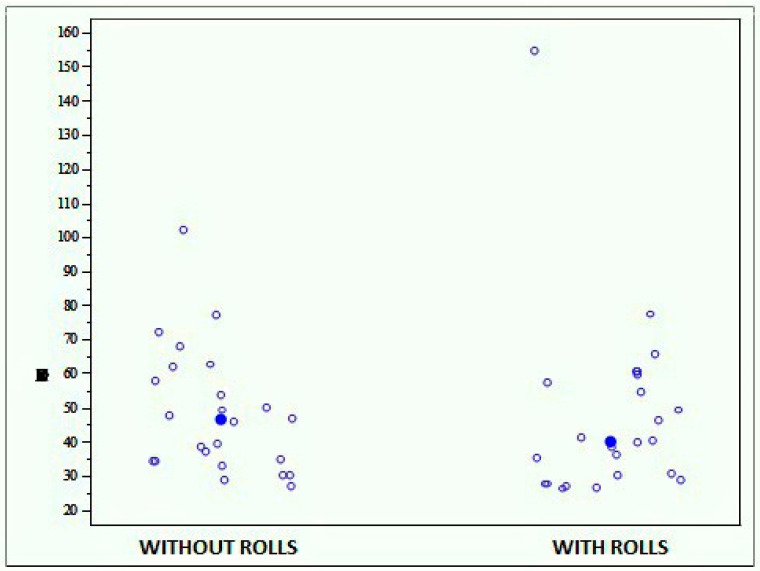
Graph showing the scores obtained with the Prokin-B device at the proprioceptive evaluation through foot-ankle circling with and without rolls.

This test consisted in the execution of three clockwise turns with the right foot and three anticlockwise turns with the left foot. The average score reported during the test performed without rolls was 48.671 (±18.432), while the one reported during the test performed with rolls was 47.819 (±27.096), showing no statistically significant differences (*p* = 0.4693) (Figure 12).

• Monopodalic stability evaluation with and without rolls

**Figure 13:**
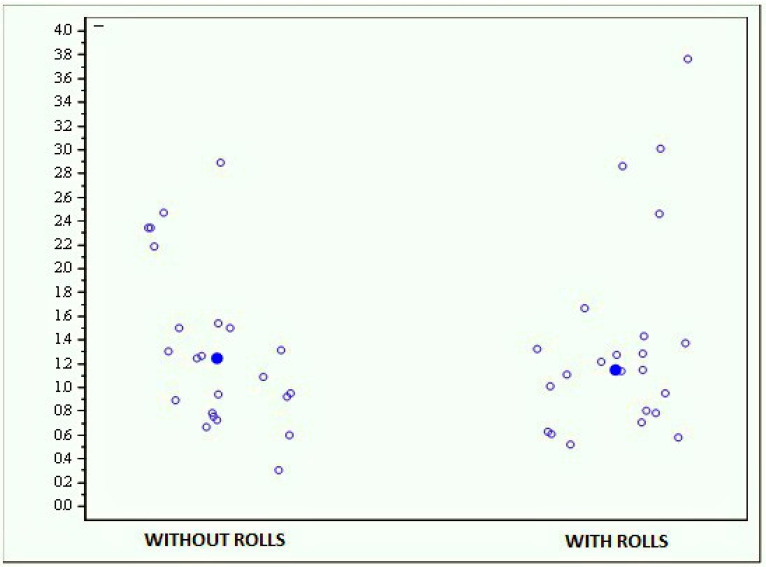
Graph showing the scores obtained with the Prokin-B device at the monopodalic stability evaluation with and without rolls.

The results obtained by the whole sample with and without rolls are quite similar (*p* = 0.7017), with an average score of 1.328 (±0.687) at the test performed without rolls and of 1.376 (±0.854) at the test performed with rolls (Figure 13).

## 4. Discussion

The aim of this study was to investigate the presence of a possible association between dental malocclusions and body posture and balance in static and dynamic conditions.

In the literature, many studies have analyzed this topic, often reporting conflicting results. Indeed, some of these articles confirm such an association, while others show no correlation.

For example, Michelotti et al. claim that there is no reason to perform occlusal and orthodontic treatment to treat or prevent postural imbalances or the alteration of spine curvatures [23]. On the contrary, Martensmeier demonstrated that patients with class I and II malocclusion exhibit a significant improvement of cervical spine curvature following orthodontic treatment [19].

None of the subjects enrolled in our work showed significant dental malocclusions. Many of them reported a recent history of receiving orthodontic corrective treatment in the past, and this can partly explain the lack of serious occlusal pathologies in the examined population.

In the present study, we used modern evaluation tools to assess posture and balance. Other studies in the literature have reported the use of the same or other similar devices, both to assess postural control in different conditions and as potential rehabilitation tools [24,25,26,27]. However, no other work has previously investigated the relationship between dental occlusion and body balance by using such modern devices.

The results we achieved from our study sample do not show a statistically significant difference between all the stabilometric evaluations performed with and without cotton rolls positioned between the dental arches, both in bipodalic and monopodalic equilibrium.

As body balance derives from the interaction between different systems, including the visual one, we also tried to investigate if the correlation between dental occlusion and neuromotor control could be modified by visual control. Anyhow, the scores obtained during the tests performed with and without cotton rolls did not change with open or closed eyes. These results are in contrast with those obtained by Tardieu et al., who demonstrated that the presence of dental contact worsens stabilometric control only when visual control is absent [20].

## 5. Conclusions

This study showed no statistically significant correlations between dental occlusion and neuromuscular control in young and healthy individuals. Such an absence of association was observed in all the conditions we tested (stable and unstable platforms, open or closed eyes, bipodalic and monopodalic support).

The main limitations of this study are represented by the limited sample and the absence of a radiological examination to support clinical evaluation.

However, we suggest that the tests we performed and the high-quality evaluation tools we employed in this study may be useful for future research to evaluate subjects with malocclusion before and after orthodontic treatment, even athletes during the execution of specific sporting gestures.
